# Investigation of Viscoelastic Guided Wave Properties in Anisotropic Laminated Composites Using a Legendre Orthogonal Polynomials Expansion–Assisted Viscoelastodynamic Model

**DOI:** 10.3390/polym16121638

**Published:** 2024-06-10

**Authors:** Hongye Liu, Ziqi Huang, Zhuang Yin, Maoxun Sun, Luyu Bo, Teng Li, Zhenhua Tian

**Affiliations:** 1 School of Optical–Electrical and Computer Engineering, University of Shanghai for Science and Technology, 580 Jungong Road, Shanghai 200093, China; liuhongye@usst.edu.cn (H.L.); sunmaoxun@usst.edu.cn (M.S.); 2 Department of Mechanical Engineering, Virginia Polytechnic Institute and State University, 1145 Perry Street, Blacksburg, VA 24061, USA

**Keywords:** carbon–fiber–reinforced composites, viscoelastodynamics, guided waves, Legendre orthogonal polynomial, dispersion curves

## Abstract

This study investigates viscoelastic guided wave properties (e.g., complex–wavenumber–, phase–velocity–, and attenuation–frequency relations) for multiple modes, including different orders of antisymmetric, symmetric, and shear horizontal modes in viscoelastic anisotropic laminated composites. To obtain those frequency–dependent relations, a guided wave characteristic equation is formulated based on a Legendre orthogonal polynomials expansion (LOPE)–assisted viscoelastodynamic model, which fuses the hysteretic viscoelastic model–based wave dynamics and the LOPE–based mode shape approximation. Then, the complex–wavenumber–frequency solutions are obtained by solving the characteristic equation using an improved root–finding algorithm, which leverages coefficient matrix determinant ratios and our proposed local tracking windows. To trace the solutions on the dispersion curves of different wave modes and avoid curve–tracing misalignment in regions with phase–velocity curve crossing, we presented a curve–tracing strategy considering wave attenuation. With the LOPE–assisted viscoelastodynamic model, the effects of material viscosity and fiber orientation on different guided wave modes are investigated for unidirectional carbon–fiber–reinforced composites. The results show that the viscosity in the hysteresis model mainly affects the frequency–dependent attenuation of viscoelastic guided waves, while the fiber orientation influences both the phase–velocity and attenuation curves. We expect the theoretical work in this study to facilitate the development of guided wave–based techniques for the NDT and SHM of viscoelastic anisotropic laminated composites.

## 1. Introduction

In the case of non–destructive testing (NDT) [1,2] and structural health monitoring (SHM) [2,3], guided waves are widely used due to their features, including long propagation distances along different waveguides, such as plates, pipes, and bars with arbitrary cross–sections, as well as the high sensitivity to different types of damages, such as cracks, corrosion, and delamination. For the development of novel multifunctional materials, as well as NDT and SHM techniques for viscoelastic anisotropic laminated composites, it is of high importance to investigate viscoelastodynamics, such as propagating and non–propagating guided waves, for waveguides with anisotropic viscoelastic properties [4]. Fiber–reinforced composites have high strength–to–weight ratios and show great potential for developing lightweight structures for aerospace and automobile applications. However, these engineered materials can have internal defects, such as delamination, fiber breaking, and matrix cracking, during their production and service [5,6,7]. The inherent material anisotropy and unique internal defects make the NDT and SHM of fiber–reinforced composites very different from the inspection and monitoring of isotropic metallic structures.

Due to the frequency–dependent wave properties (i.e., dispersion) and multi–mode features, guided wave propagation is quite complex. Especially for anisotropic viscoelastic waveguides, on the one hand, material anisotropy leads to direction–dependent wave properties. On the other hand, the consideration of material viscosity in a viscoelastic waveguide leads to many non–propagating wave modes carrying large imaginary wavenumbers, as well as frequency–dependent attenuation for propagating modes. The material viscosity also extends the solution space of the guided wave characteristic equation to the multi–dimensional frequency–complex–wavenumber space, further raising challenges, such as how to efficiently find roots in that multi–dimensional space, as well as how to correctly classify those roots to different wave modes for obtaining their dispersion curves (also known as curve tracing). If these challenges are not properly addressed, multiple undesired scenarios may happen, such as missing roots when multiple roots are very close to each other in the solution space, as well as curve–tracing misalignment in regions where phase–velocity curves of different modes cross each other (i.e., dispersion curve crossing). The complexity of guided waves in viscoelastic anisotropic waveguides, such as viscoelastic, anisotropic, laminated carbon–fiber reinforced polymer (CFRP) composites, leads to challenges in developing analytical models for investigating guided wave properties in those waveguides. Hence, an efficient and robust approach is highly desired, for studying viscoelastic guided waves in the anisotropic waveguides with viscoelastic properties considered, such as viscoelastic anisotropic laminated composites.

Motivated by the NDT and SHM of industrial structures with viscoelastic coatings (e.g., thick corrosion–resistant coatings), researchers began to investigate the properties of viscoelastic guided waves by fusing partial wave superposition techniques, global and transfer matrix methods for deriving wave characteristic equations, and different viscoelastic models, such as the Kelvin–Voigt, Maxwell, and Hysteretic viscoelastic models. For example, Pan et al. [8] investigated guided waves in a steel plate with a thick viscoelastic coating and found that the coating layer introduced additional dispersive modes by establishing a model considering multiple damping factors in addition to the Lamé constants. With the transfer matrix method, Simonetti [9] analyzed the effects of attenuative coatings on the dispersion characteristics of guided waves in coated metallic plates. By using the global matrix method, Barshinger and Rose [10] established a characteristic equation considering frequency–dependent complex Lamé constants for an elastic cylinder with a viscoelastic coating. In addition to structures with viscoelastic coatings, Huber [11] calculated the guided wave–dispersion curves for fluid–loaded viscoelastic composites consisting of up to 400 layers by using an open–access software (Dispersion Calculator v2.4) that employed the global matrix method and the hysteretic viscoelastic model. Although the transfer and global matrix methods can be used for solving guided wave–dispersion relations, these methods are not suitable for complex–shaped waveguides. Additionally, the transfer matrix method is unstable at high frequencies [9].

Since the analysis of guided waves in waveguides with complex cross–sections poses a challenge to methods based on transfer and global matrixes, numerical techniques, such as spectral element, boundary element, and finite–element–assisted approaches, have been developed. Based on the spectral finite–element method, Marzani [12] investigated the transient responses of attenuative guided waves in damped cylinders, and Luo et al. [13] studied the propagation of guided waves in layered viscoelastic film materials. By establishing boundary finite–element–based models considering the anti–Zener and fractional Zener viscoelastic models, Bause et al. [14] simulated the transient responses of guided waves in viscoelastic waveguides. Hayashi et al. [15] established a semi–analytical finite–element (SAFE) method to obtain the phase– and group–velocity dispersion curves for bars with arbitrary cross–sections. Bartoli et al. [16] introduced the hysteretic and Kelvin–Voigt viscoelastic models to the SAFE approach to study viscoelastic guided waves. Mazzotti et al. [17] extended the SAFE method for studying guided waves in prestressed viscoelastic waveguides. Taking advantage of the SAFE approach, they further investigated the propagation of guided waves in a viscoelastic waveguide with an arbitrary cross–section embedded in a viscoelastic isotropic unbounded medium [18], as well as obtained the dispersion curves of guided waves in thin–walled waveguides in contact with fluids [19]. Considering both material anisotropy and viscoelasticity, Castaings and Hosten [20] established a SAFE model to obtain dispersion curves and through–thickness mode shapes for guided waves in sandwich structures made of viscoelastic anisotropic composite materials.

While methods assisted by the aforementioned numerical techniques show good feasibility for analyzing guided waves in complex–shaped waveguides, they are subjected to an inherent contradiction between computational cost and solution accuracy that typically depends on the discretization resolution [21]. To increase the computational efficiency, Torres–Arredondo and Fritzen [22] leveraged the higher–order plate theory to find the dispersion solutions for viscoelastic fiber–reinforced composites, and their method leveraged the hysteretic and Kelvin–Voigt models for introducing the viscoelastic effect. Based on the idea of using the field variable’s orthogonal basis expansion for solving wave dynamic problems, Yang and Yu [23] proposed an approach fusing the LOPE and the Kelvin–Voigt viscoelastic model to investigate shear horizontal waves in hollow cylinders made of functionally graded materials. Dahmen et al. [24] and Othmani et al. [25] leveraged LOPE to formulate models for studying guided waves in viscoelastic composites, while not analyzing wave mode shapes or addressing the issue of tracing the dispersion curves for multiple wave modes. By leveraging the LOPE, linear three–dimensional (3D) elasticity theory, and mechanics of incremental deformation theory, Liu et al. [26] presented an approach to characterize guided wave propagation along the non–principal symmetry axes of pre–stressed anisotropic composite laminas. Building upon the mechanics of incremental deformation theory and the Kelvin–Voight model, Li et al. [27] proposed a recursive LOPE method to find the dispersion curves of guided waves in prestressed bolts.

Unlike the literature studies [24,25,26], in which the transformation of a wave–dispersion problem into an eigenvalue problem makes them susceptible to the curve–tracing misalignment issue, this paper presents an approach that takes advantage of the LOPE, the hysteretic viscoelastic model, and unique root–finding and curve–tracing strategies to formulate the guided wave characteristic equation and obtain the frequency–dependent properties of guided waves for viscoelastic anisotropic laminated composites. Particularly, our wave characteristic equation is formulated using a LOPE–assisted viscoelastodynamic model, which takes advantage of the hysteretic viscoelastic model–based viscoelastodynamics and the LOPE–based wave mode shape approximation. To solve the characteristic equation, we have introduced an improved root–finding algorithm, building upon the coefficient matrix determinant ratios and our proposed local tracking windows. To trace the solutions on the different modes’ dispersion curves, we have proposed a curve–tracing strategy considering wave attenuation. It is worth noting that our root–finding and curve–tracing strategies effectively address the issues, including losing roots on different solution branches close to each other and curve–tracing misalignment in regions where the phase–velocity curves of different modes cross each other. Moreover, based on the LOPE–assisted viscoelastodynamic model, we have investigated viscoelastic guided wave properties (such as complex–wavenumber–, phase–velocity–, and attenuation–frequency relations) for multiple guided wave modes, including different orders of antisymmetric, symmetric, and shear horizontal modes in viscoelastic anisotropic laminated composites. We have also unveiled the effects of material viscosity and fiber orientation on the frequency–dependent properties of different guided wave modes for unidirectional CFRP laminated composites. The rest of this paper is structured as follows. Section 2 presents the LOPE–assisted viscoelastodynamic model and formulated the characteristic equation for viscoelastic guided waves. Section 3 presents the root–finding and curve–tracing strategies; multiple case studies are given in Section 4. The concluding remarks are presented in Section 5.

## 2. A LOPE–Based Model for Viscoelastic Guided Waves

**Figure 1:**
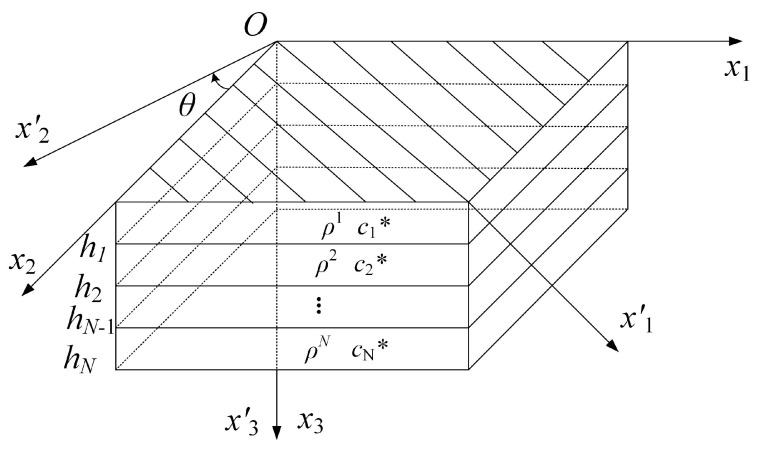
Schematic diagram showing the model setup for a viscoelastic anisotropic fiber–reinforced laminated composite. This schematic also shows a global coordinate system *O*–*x*_1_*x*_2_*x*_3_ for the laminated composite and a local coordinate system *o*–*x*_1_’*x*_2_’*x*_3_’ for the top lamina with *x*_1_’ along the fiber direction. In this schematic, the point *O* coincides with *o*. In fact, these two points can be at different positions on the top surface of the laminate.

Consider an anisotropic viscoelastic laminated composite with infinite in–plane lengths and stress–free conditions on the top and bottom boundaries, as illustrated in Figure 1. The thickness of the *n^th^* (*n* = 1, 2, …, *N*) ply is *d_n_* = *h_n_* − *h_n_*_−1_, where *h_n_* is the height from the bottom of the *n^th^* ply to the top of the first ply. The density of the *n^th^* ply is denoted as $\rho^{\left(n\right)}$. To consider the viscoelastic effect, our approach uses the hysteretic viscoelastic model [13,21], where the viscoelastic stiffness matrix has frequency–independent complex values. The viscoelastic stiffness matrix for the constitutive equation in the global coordinate system *O*–*x*_1_*x*_2_*x*_3_ (illustrated in Figure 1) for the *n^th^* lamina can be expressed as:

(1) $$c_{ijkl}^{*\left(n\right)}=c_{ijkl}^{\left(n\right)}+i\mu_{ijkl}^{\left(n\right)}$$

where $c_{ijkl}^{\left(n\right)}$ and $\mu_{ijkl}^{\left(n\right)}$ represent the elastic stiffness and viscous matrices. For a fiber–reinforced laminated composite, different laminas can have different orientations. Once we know the fiber orientation of a lamina, $c_{ijkl}^{*\left(n\right)}$ can be obtained using the matrix $c_{ijkl}^{’*}$ for the lamina’s local coordinate system *o*–*x*_1_’*x*_2_’*x*_3_’ through tensor rotation [28].

For a laminated composite with *N* layers, its material properties change along the *x*_3_–direction of the global coordinate system. To mathematically describe the properties of the composites with all the layers considered, the viscoelastic stiffness matrix $c_{ijkl}^{*}$ and density ρ can be expressed as:

(2) $$c_{ijkl}^{*}=\sum_{n=1}^{N}c_{ijkl}^{*\left(n\right)}\pi_{h_{n-1},h_{n}}\left(x_{3}\right)$$

(3) $$\rho =\sum_{n=1}^{N}\rho^{\left(n\right)}\pi_{h_{n-1},h_{n}}\left(x_{3}\right)$$

where $\pi_{h_{n-1},h_{n}}\left(x_{3}\right)$ is a rectangular window function:

(4) $$\pi_{h_{n-1},h_{n}}\left(x_{3}\right)=\left\{\begin{array}{c} 1,\;\;\;\;h_{n-1}<x_{3}<h_{n}\\ 0,\;\;\;\;\;\;\;\;elsewhere \end{array}\right.$$

To derive the characteristic equation for guided waves, one typically needs to fuse the equation of motion, stress–strain constitutive equation, strain–displacement relations, and general wave displacement solutions. Without considering the body–force term, the equation of motion for a viscoelastic laminated composite can be expressed as [29]:

(5) $$\begin{array}{c} \frac{\partial T_{11}}{\partial x_{1}}+\frac{\partial T_{12}}{\partial x_{2}}+\frac{\partial T_{13}}{\partial x_{3}}=\rho \frac{\partial^{2}u_{1}}{\partial t^{2}}\\ \frac{\partial T_{21}}{\partial x_{1}}+\frac{\partial T_{22}}{\partial x_{2}}+\frac{\partial T_{23}}{\partial x_{3}}=\rho \frac{\partial^{2}u_{2}}{\partial t^{2}}\\ \frac{\partial T_{31}}{\partial x_{1}}+\frac{\partial T_{32}}{\partial x_{2}}+\frac{\partial T_{33}}{\partial x_{3}}=\rho \frac{\partial^{2}u_{3}}{\partial t^{2}} \end{array}$$

where $T_{ij}$ represents the stress tensor and $u_{i}$ stands for a displacement component. According to the generalized Hooke’s law, the stress–strain constitutive equation is:

(6) $$\left\{\begin{array}{c} T_{11}\\ T_{22}\\ T_{33}\\ T_{23}\\ T_{13}\\ T_{12} \end{array}\right\}=C^{*}\left\{\begin{array}{c} S_{11}\\ S_{22}\\ S_{33}\\ 2S_{23}\\ 2S_{13}\\ 2S_{12} \end{array}\right\}$$

where $C^{*}$ is the viscoelastic stiffness matrix. As guided waves have small deformations, the strain–displacement relation in the Cartesian coordinate system can be expressed as:

(7) $$S_{i,j}=\frac{1}{2}\left(\frac{\partial u_{i}}{\partial x_{j}}+\frac{\partial u_{j}}{\partial x_{i}}\right)$$

Assuming that the laminated composite is large enough in the direction of $x_{2}$ to satisfy the plane strain condition, the displacement of a wave propagating in the *x*_1_ direction is independent of $x_{2}$. Therefore, for guided waves propagating in the $x_{1}$ direction, at a position ($x_{1}$, $x_{2}$, $x_{3}$), the general solutions of 3D wave displacements $u_{1}$, $u_{2}$, and $u_{3}$ along the $x_{1}$−, $x_{2}$−, and $x_{3}$ axes can be expressed as [29]:

(8) $$\begin{array}{c} u_{1}\left(x_{1},x_{2},x_{3},t\right)=U\left(x_{3}\right)e^{i\left(kx_{1}-\omega t\right)}\\ u_{2}\left(x_{1},x_{2},x_{3},t\right)=V\left(x_{3}\right)e^{i\left(kx_{1}-\omega t\right)}\\ u_{3}\left(x_{1},x_{2},x_{3},t\right)=W\left(x_{3}\right)e^{i\left(kx_{1}-\omega t\right)} \end{array}$$

where $U\left(x_{3}\right)$, $V\left(x_{3}\right)$, and $W\left(x_{3}\right)$ represent the wave mode shape displacements in the directions of $x_{1}$, $x_{2}$, and $x_{3}$, respectively. *k* is a complex wavenumber corresponding to the wave propagation direction $x_{1}$, and ω is the angular frequency.

By substituting Equations (6)–(8) into the equation of motion, we can derive the following wave equations:

(9) $$\begin{array}{c} {}[C_{55}^{*}U^{\prime \prime }\left(x_{3}\right)-C_{11}^{*}k^{2}U\left(x_{3}\right)+C_{45}^{*}V^{\prime \prime }\left(x_{3}\right)-C_{16}^{*}k^{2}V\left(x_{3}\right)+(C_{13}^{*}\\ +C_{55}^{*})\left(ik\right)W^{\prime }\left(x_{3}\right)]\pi_{h_{0},h_{n}}\left(x_{3}\right)+[C_{55}^{*}U^{\prime }\left(x_{3}\right)+C_{45}^{*}V^{\prime }\left(x_{3}\right)+C_{55}^{*}\left(ik\right)\\ W\left(x_{3}\right)]{\pi^{\prime }}_{h_{0},h_{n}}\left(x_{3}\right)=-\rho \omega^{2}U\left(x_{3}\right)\pi_{h_{0},h_{n}}\left(x_{3}\right) \end{array}$$

(10) $$\begin{array}{c} {}[C_{45}^{*}U^{\prime \prime }\left(x_{3}\right)-C_{16}^{*}k^{2}U\left(x_{3}\right)+C_{44}^{*}V^{\prime \prime }\left(x_{3}\right)-C_{66}^{*}k^{2}V\left(x_{3}\right)+\\ \left(C_{45}^{*}+C_{36}^{*}\right)\left(ik\right)W^{\prime }\left(x_{3}\right)]\pi_{h_{0},h_{n}}\left(x_{3}\right)+[C_{45}^{*}U^{\prime }\left(x_{3}\right)+C_{44}^{*}V^{\prime }\left(x_{3}\right)\\ +C_{45}^{*}\left(ik\right)W\left(x_{3}\right)]{\pi^{\prime }}_{h_{0},h_{n}}\left(x_{3}\right)=-\rho \omega^{2}V\left(x_{3}\right)\pi_{h_{0},h_{n}}\left(x_{3}\right) \end{array}$$

(11) $$\begin{array}{c} {}[\left(C_{13}^{*}+C_{55}^{*}\right)\left(ik\right)U^{\prime }\left(x_{3}\right)+\left(C_{36}^{*}+C_{45}^{*}\right)\left(ik\right)V^{\prime }\left(x_{3}\right)+C_{33}^{*}W^{\prime \prime }\left(x_{3}\right)\\ -C_{55}^{*}k^{2}W\left(x_{3}\right)]\pi_{h_{0},h_{n}}\left(x_{3}\right)+[C_{13}^{*}\left(ik\right)U\left(x_{3}\right)+C_{36}^{*}\left(ik\right)V\left(x_{3}\right)\\ +C_{33}^{*}W^{\prime }\left(x_{3}\right)]{\pi^{\prime }}_{h_{0},h_{n}}\left(x_{3}\right)=-\rho \omega^{2}W\left(x_{3}\right)\pi_{h_{0},h_{n}}\left(x_{3}\right) \end{array}$$

In these equations, the mode shape displacements *U*$\left(x_{3}\right)$, $V\left(x_{3}\right)$, and $W\left(x_{3}\right)$ can be expanded with complete and orthogonal Legendre polynomials, as follows [26,27]:

(12) $$\begin{array}{c} U\left(x_{3}\right)=\sum_{m=0}^{\infty }p_{m}^{1}Q_{m}\left(x_{3}\right)\\ V\left(x_{3}\right)=\sum_{m=0}^{\infty }p_{m}^{2}Q_{m}\left(x_{3}\right)\\ W\left(x_{3}\right)=\sum_{m=0}^{\infty }p_{m}^{3}Q_{m}\left(x_{3}\right) \end{array}$$

where $p_{m}^{i}$ is the expansion coefficient, and $Q_{m}\left(x_{3}\right)$ is a normalized *m^th^*–order Legendre orthogonal polynomial expressed as:

(13) $$Q_{m}\left(x_{3}\right)=\sqrt{\frac{2m+1}{h_{N}}}P_{m}\left(\frac{2x_{3}}{h_{N}}-1\right)$$

where *P_m_* is the *m^th^*–order Legendre polynomial. In practice, the order *m* is truncated to a finite value *M*, which can be determined through convergence analysis.

Equations (9)–(11) are multiplied by $Q_{j}^{*}\left(x_{3}\right)$ with *j* running from zero to M and then integrated over $x_{3}$ from zero to $h_{N}$. Further considering the orthogonality of the polynomials $Q_{m}\left(x_{3}\right)$, we can obtain following equation set:

(14) $$\left\{\begin{array}{c} \left(A_{11}^{m,j}+\omega^{2}M_{m}^{j}\right)p_{m}^{1}+A_{12}^{m,j}p_{m}^{2}+A_{13}^{m,j}p_{m}^{3}=0\\ A_{21}^{m,j}p_{m}^{1}+\left(A_{22}^{m,j}+\omega^{2}M_{m}^{j}\right)p_{m}^{2}+A_{23}^{m,j}p_{m}^{3}=0\\ A_{31}^{m,j}p_{m}^{1}+A_{32}^{m,j}p_{m}^{2}+\left(A_{33}^{m,j}+\omega^{2}M_{m}^{j}\right)p_{m}^{3}=0 \end{array}\right.$$

where $A_{\alpha \beta }^{m,j}\left(\alpha ,\beta =1,2,3\right)$ and $M_{m}^{j}$ are coefficients stemming from Equations (9)–(11). For detailed information, please refer to Appendix A. The non–zero solutions of Equation (14) can only exist when the determinant of the coefficient matrix for $p_{m}^{i}$ equals zero. This leads to a characteristic equation expressed as:

(15) $$\Omega \left(\omega ,k\right)=\left|\begin{array}{ccc} A_{11}^{m,j}+\omega^{2}M_{m}^{j} & A_{12}^{m,j} & A_{13}^{m,j}\\ A_{21}^{m,j} & A_{22}^{m,j}+\omega^{2}M_{m}^{j} & A_{23}^{m,j}\\ A_{31}^{m,j} & A_{32}^{m,j} & A_{33}^{m,j}+\omega^{2}M_{m}^{j} \end{array}\right|=0$$

The solutions of the characteristic equation provide the relationship between angular frequency *ω* and complex wavenumber *k*. Then, we can obtain the phase velocity with $C_{p}=\omega /Re\left(k\right)$ and analyze the wave attenuation with $Im\left(k\right)$. Here, $Re\left(k\right)$ and $Im\left(k\right)$ represent the real and imaginary parts of a complex wavenumber.

## 3. Root–Finding and Curve–Tracing Strategies

Distinguishing from previous studies [24,25,26], which transform the problem of finding an anisotropic laminated composite’s wave–dispersion relation into an eigenvalue problem, this study directly solves the wave–dispersion characteristic equation derived from a LOPE–based method by using an improved root–finding algorithm leveraging coefficient matrix determinant ratios and our proposed local tracking intervals. Although the bisection and Newton–Raphson methods, common iterative root–finding algorithms, have been shown to solve dispersion characteristic equations for elastic materials, however, they are difficult to implement for solving the characteristic equations for viscoelastic materials. The introduction of viscosity makes the dispersion characteristic equation have complex wavenumbers, and this leads to challenges in using the traditional bisection method for root finding in the frequency–complex–wavenumber space. The Newton–Raphson method, which requires solving the inverse matrix for the objective function’s Hessian matrix, is computationally intensive. A previous study [30], whose wave characteristic equation was derived using partial wave superposition, showed that the ratio of coefficient matrix determinants could be quickly obtained to solve the dispersion relation for a viscoelastic plate. In this study, we solved the wave–dispersion characteristic equation derived from a LOPE–based approach by using an improved determinant ratio–based root–finding approach with our proposed local tracking intervals. Moreover, an improved curve–tracing strategy that takes advantage of wave–attenuation curves is presented for classifying characteristic equation solutions to different modes, in order to address the curve–tracing misalignment issue that usually happens when only using the phase–velocity–frequency data for curve tracing.

### 3.1. Root–Finding Algorithm Based on Determinant Ratios

Considering a general univariate equation $\Omega \left(x\right)=0$, finding the roots of this equation is equivalent to solving the relation $\left|\Omega \left(x\right)\right|=0$. To find the local minima of the determinant function $\left|\Omega \left(x\right)\right|$, the initial search intervals can be divided equally into many tiny segments with discrete nodes. By evaluating the values of the determinant $\left|\Omega \left(x\right)\right|$ at the nodes on either side of each tiny segment, the local minima in the searched domain can be obtained.

**Figure 2:**
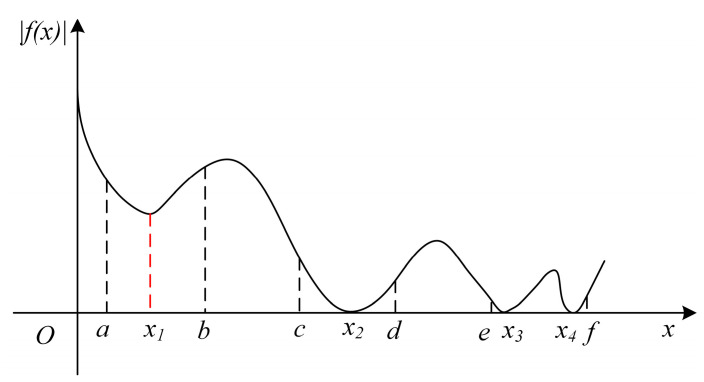
(Color online) A graph for illustrating the determinant function $\left|f\left(x\right)\right|$ and three representative cases, including a section [*a*, *b*] containing a local minimum with |*f*(*x*_1_)| > 0 at *x*_1_, a section [*c*, *d*] containing a local minimum with |*f*(*x*_2_)| = 0 at *x*_2_, and a section [*e*, *f*] having two local minima with |*f*(*x*_3_)| = |*f*(*x*_4_)| = 0 and at *x*_3_ and *x*_4_.

All the local minima can be divided into two categories: those that satisfy $\left|\Omega \left(x\right)\right|=0$, e.g., the point $x_{2}$ within an interval $\left[c,d\right]$ shown in Figure 2, and those that do not, e.g., the point $x_{1}$ within an interval $\left[a,b\right]$. Only the minima that satisfy $\left|\Omega \left(x\right)\right|=0$ are the solutions of the original function $\Omega \left(x\right)=0$. For example, the point $x_{1}$ in Figure 2 is not a solution, as the determinant $\left|\Omega \left(x_{1}\right)\right|$ is greater than zero. Hence, there should be a finite positive number P satisfying the following determinant ratios.

(16) $$\frac{\left|\Omega \left(a\right)\right|}{\left|\Omega \left(x_{1}\right)\right|}<P$$

(17) $$\frac{\left|\Omega \left(b\right)\right|}{\left|\Omega \left(x_{1}\right)\right|}<P$$

On the contrary, for the point $x_{2}$ within an interval $\left[c,d\right]$, as $\left|\Omega \left(x_{2}\right)\right|=0$, the determinant ratios should be greater than P, as expressed in the following relations,

(18) $$\frac{\left|\Omega \left(c\right)\right|}{\left|\Omega \left(x_{2}\right)\right|}>P$$

(19) $$\frac{\left|\Omega \left(d\right)\right|}{\left|\Omega \left(x_{2}\right)\right|}>P$$

The finite positive number P can be considered as a threshold for finding the solutions of $\left|\Omega \left(x\right)\right|=0$. When an interval has multiple solutions, e.g., an interval $\left[e,f\right]$ with two solutions, $x_{3}$ and $x_{4}$, illustrated in Figure 2, the aforementioned root–finding approach may lose a root. To circumvent this issue, a small interval is required, and it should be less than the distance between any two solutions.

### 3.2. Improved Root–Finding Algorithm

**Figure 3:**
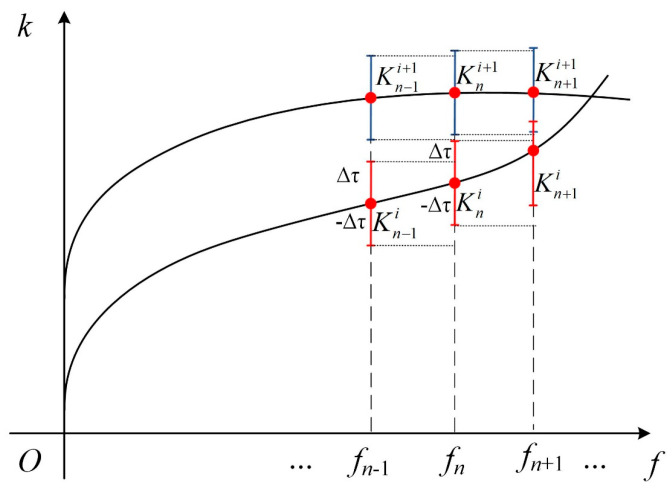
(Color online) Schematic diagram for illustrating the root–finding algorithm for obtaining frequency–wavenumber solutions for the LOPE–based guided wave characteristic equation.

When extending the aforementioned univariate problem’s root–finding algorithm to a bivariate scenario, if the used search interval is too large, it will lead to root loss. Nevertheless, if the search interval is too small, it will result in high computational cost. In order to elaborate on this issue, Figure 3 gives a schematic for the two–dimensional (2D) root–finding process for solving the characteristic equation $\left|\Omega \left(k,\;\omega =\frac{f}{2\pi }\right)\right|=0$. The two solid lines in Figure 3 represent two frequency–wavenumber curves (i.e., solutions) of the equation. After selecting the frequency interval Δf, a series of discrete frequency points can then be obtained in the frequency domain, where $f_{n}$ denotes the *n*^th^ frequency for root finding. Assume that the wavenumber *K^i^_n_* is the *i*^th^ discrete point along the wavenumber axis at the frequency $f_{n}$, and the point ($f_{n}$, *K^i^_n_*) in the frequency–wavenumber space is a solution for the characteristic equation. At the frequency $f_{n+1}$, the wavenumber solutions can be obtained by applying the univariate root–finding approach along the wavenumber axis. However, during the iterative search process, if the seeking increment $\Delta K_{n+1}^{i}$ is too large, e.g., larger than the distance between the wavenumbers $K_{n+1}^{i+1}$ and $K_{n+1}^{i}$ on two solution curves, the situation of missing a root would occur. If a very small wavenumber interval is used for the iterative process, the time consumption will significantly increase.

The limitation of the previous root–finding algorithm is improved by employing a search strategy with a local tracking interval. First, an initial root finding is performed to obtain the starting frequency–wavenumber point, for example, a solution at (*f_n−_*_1_, $K_{n-1}^{i}$) for the characteristic equation at an initial frequency of *f_n−_*_1_. Then, the next frequency *f_n_* is allocated with *f_n−_*_1_ + ∆*f*, where ∆*f* is a small frequency interval. Finally, the wavenumber *K^i^_n_* at the frequency *f_n_* can be found by searching a local tracking interval $\left[K_{n-1}^{i}-\Delta \tau ,K_{n-1}^{i}+\Delta \tau \right]$, which is centered at the wavenumber $K_{n-1}^{i}$ obtained from the last searching step at the frequency *f*_*n*−1_. By continuing this searching process, all frequency–wavenumber solutions in a predefined searching domain can be obtained gradually.

### 3.3. Wave–Attenuation–Assisted Curve Tracing

**Figure 4:**
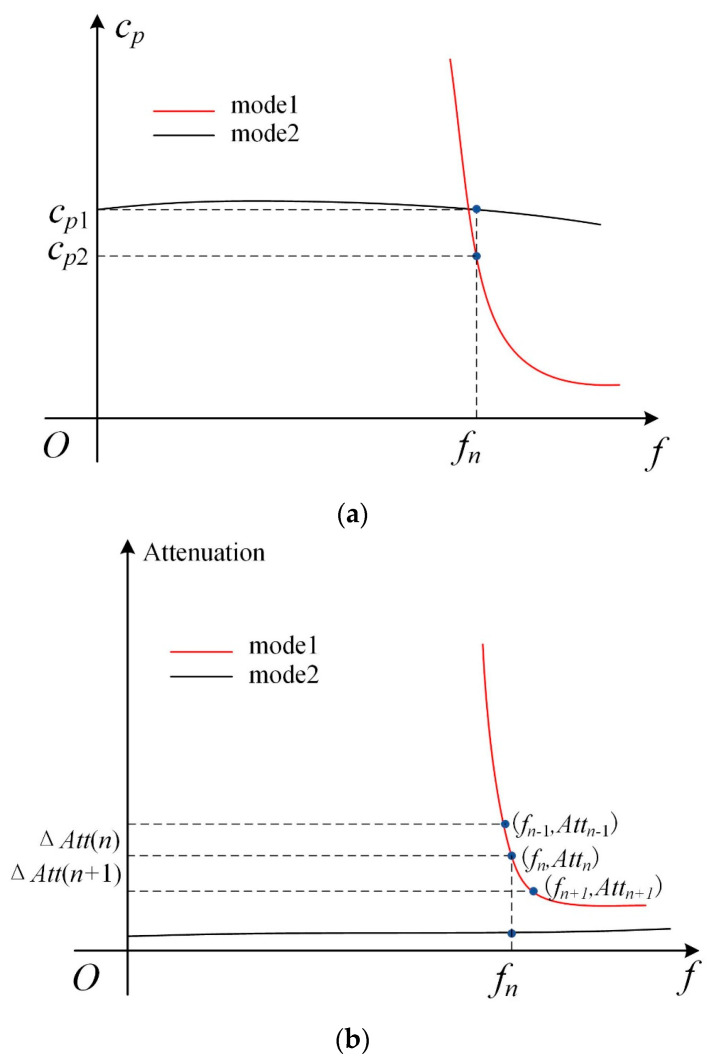
(Color online) Schematic diagram for illustrating the curve–tracing approach with (**a**) the phase–velocity dispersion curves having an intersection and (**b**) the attenuation curves for two guided wave modes.

To address the issue of curve–tracing misalignment in the region with multiple phase–velocity dispersion curves crossing or being close to each other, we presented a curve–tracing strategy based on both the phase–velocity and attenuation curves. Figure 4 displays a schematic with both the phase–velocity and attenuation curves for two modes to illustrate the curve–tracing strategy. When the phase–velocity curves of the two modes cross or are close to each other, there could be a misalignment issue for regions before and after the intersection, if only using the phase–velocity data for curving tracing. To address this issue, we also used the attenuation curves, as the attenuation values for the two modes are different at a frequency where the phase–velocity curves have an intersection.

Relying on the aforementioned feature, a curve–tracing strategy can be automatically implemented. For example, to determine whether the data point $\left(f_{n},C_{p2}\right)$ on the phase–velocity curve belongs to mode 1, two adjacent frequencies, $f_{n-1}$ = $f_{n}-\Delta f$ and $f_{n+1}$ = $f_{n}+\Delta f$ at the left and right sides of the frequency $f_{n}$, are identified at first. Then, the attenuation values at the three frequencies, $f_{n-1}$, $f_{n}$*,* and $f_{n+1}$, are computed to obtain the frequency–attenuation data ($f_{n-1}$, $Att_{n-1}$), ($f_{n}$, $Att_{n}$), and ($f_{n+1}$, $Att_{n+1}$). Finally, the attenuation differences $\Delta Att\left(n\right)$ = $Att_{n}-Att_{n-1}$ and $\Delta Att\left(n+1\right)=Att_{n+1}-Att_{n}$, as well as the residual ratio $f_{res}\left(n\right)=\Delta Att\left(n+1\right)/\Delta Att\left(n\right)$ are calculated. Therefore, if the data point $\left(f_{n},C_{p2}\right)$ belongs to mode 1, the residual ratio $f_{res}\left(n\right)$ should be very close to one, as the attenuation curve is smooth. If the residual ratio is far from one, then the data $\left(f_{n},C_{p2}\right)$ should be attributed to mode 2. By performing this procedure for all the solved data, we are able to classify the solutions to obtain dispersion curves for different wave modes.

## 4. Numerical Results

### 4.1. Validation of the LOPE–Based Approach

**Figure 5:**
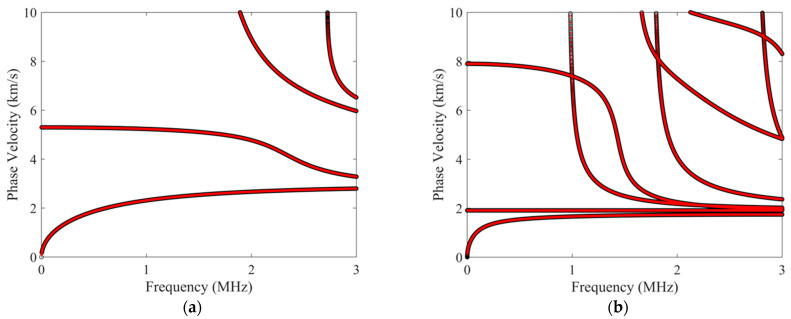
(Color online) Comparisons of the phase–velocity dispersion curves calculated by the authors’ program (the red dotted lines), with the results obtained from the “Disperse” software (the black dotted line). (**a**) Curves for an aluminum plate. (**b**) Curves for a 3–ply T300/914 CFRP composite with a layup of [0°/90°/0°].

**Figure 6:**
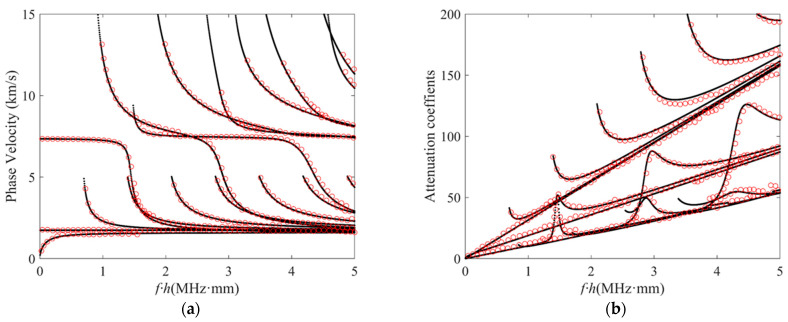
(Color online) Comparisons of (**a**) phase–velocity dispersion curves and (**b**) attenuation curves computed by the LOPE–based approach (black dots), with the results found in the literature [31] (red circles) for guided waves propagating in a 3.6 mm thick carbon epoxy plate.

Based on the dispersion characteristic equation derived from a LOPE–based approach and the aforementioned root–finding and curve–tracing strategies, customized codes in MATLAB (Mathworks) were developed to obtain the dispersion curves for guided waves in anisotropic viscoelastic laminates. To validate our approach, it was firstly applied to solve the dispersion relations for cases without considering the viscous effect, including an aluminum plate and an anisotropic laminated composite plate composed of three unidirectional T300/914 CFRP laminas in a layup of [0°/90°/0°]. The material properties for the aluminum plate and the T300/914 CFRP lamina are given in Table 1. The thickness of the aluminum plate is 1 mm, and the thickness of each lamina is 0.3 mm. Figure 5 compares the phase–velocity dispersion curves calculated by the authors’ program with the results solved by the commercial software “Disperse” based on the global matrix method (GMM) that has been experimentally validated. Both cases show that the authors’ results are in good agreement with those from the GMM–based software “Disperse”. Ref. [31] compares the LOPE–based method with the stiffness matrix method, showing small phase–velocity errors of <0.3%. Errors in this range are difficult to visually see without zooming in, especially when the phase–velocity plot covers a wide range, for example, 0 to 10 km/s in Figure 5b. In addition, a validation case was performed for a 3.6 mm thick, orthotropic carbon–epoxy plate that considers the viscous effect. The viscoelastic properties for dispersion–curve calculation are given in Table 2. Figure 6 compares the phase–velocity dispersion curves and attenuation curves computed by the authors’ approach, with the results published in a recent work [31] based on GMM. The phase–velocity dispersion curves in Figure 6a and attenuation curves in Figure 6b show that the results of our presented method and the GMM are in good agreement. The aforementioned comparisons for three different cases, including an aluminum plate, a CFRP composite, and a carbon–epoxy plate, support the correctness of our presented theoretical approach.

### 4.2. Convergence of the LOPE–Based Approach

**Figure 7:**
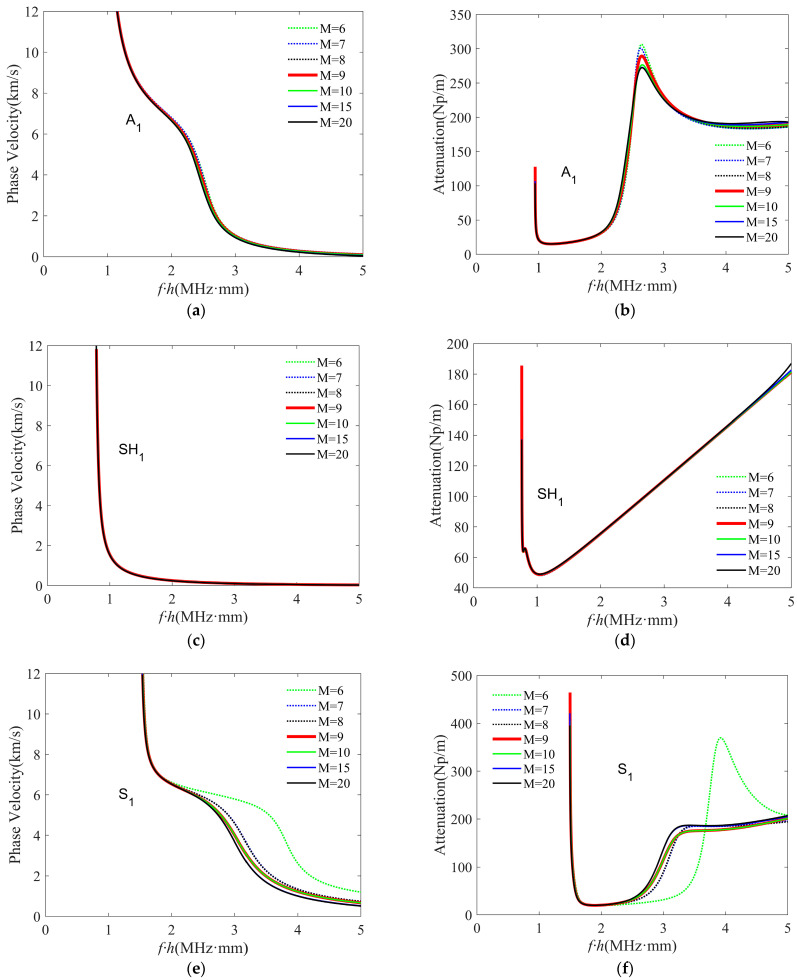
(Color online) The convergence analysis of the LOPE–based approach for (**a**,**b**) the A_1_ mode, (**c**,**d**) the SH_1_ mode, and (**e**,**f**) the S_1_ mode. The left and right columns give phase–velocity and attenuation curves, respectively. Our convergence analysis considers different truncation orders up to M = 20.

The truncation order M in the LOPE approach affects the accuracy of the solved dispersion curves, as displayed in Figure 7, including the calculated phase–velocity dispersion curves and the attenuation curves when using different truncation orders for A_1_, SH_1_, and S_1_ modes in a viscoelastic carbon–epoxy composite plate with a layup of [0°/45°/0°]. Note that we followed the method in Refs. [32,33] to label the solved modes with $S_{n}$, $A_{n}$, and $SH_{n}$ (n = 0, 1, 2, …) in this study. The material properties, including both elastic and viscous constants, of a carbon–epoxy lamina are given in Table 3. All the laminas have the same thickness of 0.3 mm.

From Figure 7, it can be observed that different modes have different convergence rates. As compared in Figure 7a,c,e, when the LOPE truncation order increases from six to seven, the phase–velocity curves of the A_1_ and SH_1_ modes slightly change, while the S_1_ mode’s phase–velocity curves change significantly. This reflects that different modes have different convergence rates. A similar conclusion can be drawn by comparing the attenuation curves. In addition, Figure 7b,e,f reveal that the phase–velocity and attenuation curves barely change in the frequency range of 0 to 2.5 MHz, when the LOPE truncation order M varies from 6 to 20. Whereas in the range with frequencies greater than 2.5 MHz, both the phase velocity and attenuation curves converge quickly with the increase of the truncation order. These observations imply that even for the same wave mode, the convergence rates are different at different frequency ranges. Considering the wave modes of interest and the frequency range for solution finding, it can be found that both the phase–velocity and attenuation curves converge well when using a truncation order M = 9. Therefore, a truncation order of nine is used for all of the following dispersion curve solving of this study.

### 4.3. Effect of Viscosity on Viscoelastic Guided Waves

For fiber–reinforced composites, the properties of viscoelastic guided waves are affected by the material properties (such as elastic stiffness and viscous matrices), material thickness, pre–stress, density, and composite layup. Therefore, factors that affect those parameters will affect the dispersion curves of viscoelastic guided waves. For example, the fiber and resin materials affect the elastic stiffness matrix, viscous matrix, and density, thus affecting the dispersion curves. This subsection focuses on investigating the effect of viscosity on viscoelastic guided waves.

**Figure 8:**
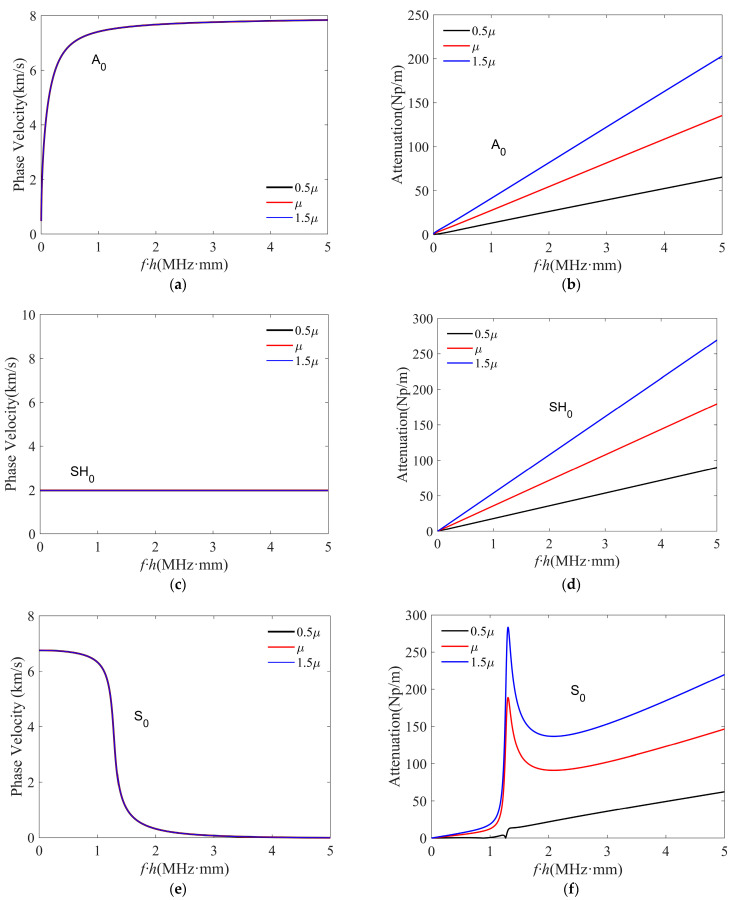
(Color online) Results for analyzing the effect of viscosity on fundamental viscoelastic guided wave modes, including (**a**,**b**) A_0_, (**c**,**d**) SH_0_, and (**e**,**f**) S_0_ modes. The left and right columns give phase–velocity and attenuation curves, respectively.

As described in Equation (1), the hysteresis model uses an imaginary part to introduce the viscous effect. For the NDT and SHM of fiber–reinforced polymers, most studies leverage the fundamental zeroth–order guided wave modes. Therefore, this section focuses on investigating the effect of viscous constants on the fundamental guided wave modes. Figure 8 gives phase–velocity and attenuation dispersion curves for three fundamental modes, A_0_, S_0_, and SH_0_, in a carbon–epoxy composite with a layup of [0°/0°/0°]. To investigate the viscous effect on the curves of different modes, we compared cases with different viscosity, including $0.5\mu_{ij}$, $\mu_{ij}$, and $1.5\mu_{ij}$, while other material properties were unchanged. The viscosity $\mu_{ij}$ can be found in Table 3.

As shown by the phase–velocity dispersion curves in Figure 8a,c,e, the phase velocities of the fundamental guided wave modes (including A_0_, S_0_, and SH_0_) barely change when the viscosity $\mu_{ij}$ increases or decreases by 50%. This implies that the phase–velocity dispersion curves solved from the hysteresis model–based characteristic equation are nearly not influenced by the viscosity change. On the other hand, the attenuation curves in Figure 8b,d,f for the three fundamental modes significantly change after changing the viscosity. For the A_0_ and SH_0_ modes, the attenuation values nearly linearly increase with the viscous effect increase and the frequency increase. In contrast, the changes of S_0_ mode attenuation values exhibit a nonlinear behavior with the viscous effect increase and the frequency increase. From Figure 8f, it can also be noticed that the S_0_ mode has a low attenuation in the low–frequency range.

### 4.4. Effects of Fiber Orientation on Viscoelastic Guided Waves

In this subsection, the effects of fiber orientation on the phase velocity and attenuation characteristics of viscoelastic guided waves are investigated. Using our approach, we calculated phase–velocity and attenuation curves for three–ply composites with different layups, including [0°/0°/0°], [0°/45°/0°], and [0°/90°/0°]. In these cases, the mid–ply orientation changes from 0° to 45° and then to 90°, while the orientations of the top and bottom plies are kept at 0°. The viscoelastic material properties for a carbon–epoxy ply are given in Table 3, and the ply thickness is 0.3 mm.

**Figure 9:**
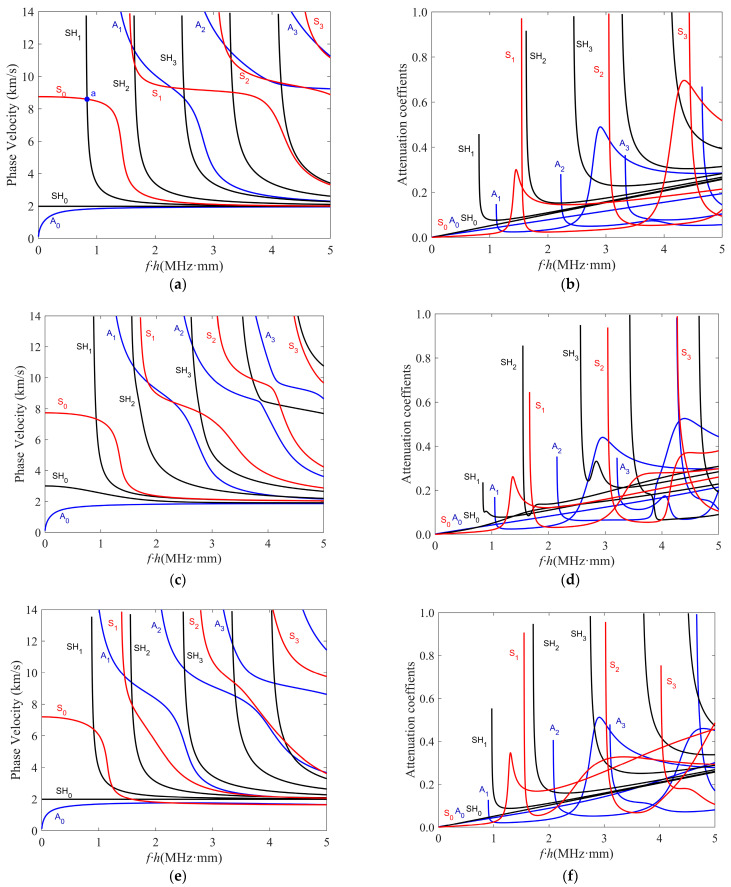
(Color online) Results for analyzing the effect of fiber orientation on guided waves. Curves for composites with layups of [0°/0°/0°], [0°/45°/0°], and [0°/90°/0°] are given in (**a**,**b**), (**c**,**d**), and (**e**,**f**), respectively. The left and right columns give phase–velocity and attenuation curves, respectively.

From the solved dispersion curves in Figure 9, we can analyze the effects of fiber orientation on the phase–velocity and attenuation curves of viscoelastic guided waves. From Figure 9a,c,e, it can be seen that some modes (i.e., high–order modes) have cut–off frequencies while others do not, namely the fundamental A_0_, SH_0_, and S_0_ modes. When the mid–ply fiber orientation increases from 0° to 90°, the phase–velocity dispersion curves of all modes gradually drift, with the high–order modes at high frequencies experiencing more significant changes. For the fundamental guided wave modes, the phase velocity of the SH_0_ mode in the low–frequency range first increases and then decreases, as the mid–ply orientation changes from 0° to 90°. The phase velocity of the S_0_ mode keeps decreasing with the increase of the mid–ply orientation angle, while the A_0_ mode phase velocity experiences smaller variations.

Similar to the circumstance of phase–velocity dispersion curves, the attenuation curves of the high–order modes also have cut–off frequencies, as shown in Figure 9b,d,f for different mid–ply orientations. It can also be noticed that the attenuation curves of high–order modes have obvious nonlinear trends with the frequency increase, while the attenuation values of the fundamental A_0_ and SH_0_ modes nearly linearly increase. The attenuation values of the S_0_ mode have near linear trends at most frequencies, except for a jump in the range from 1 to 2 MHz∙mm. The analysis in this subsection reveals that the fiber orientation affects both the phase velocity and attenuation curves of viscoelastic guided waves.

### 4.5. 3D Frequency–Wavenumber Dispersion Curves

**Figure 10:**
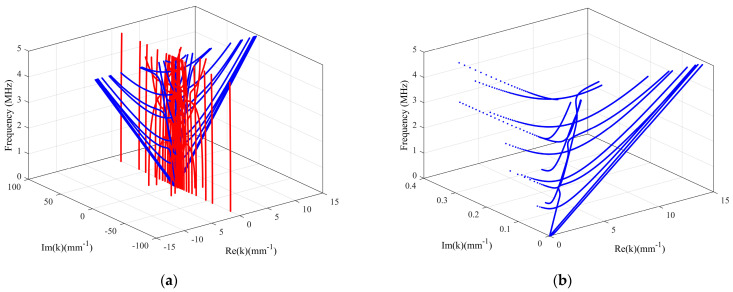
(Color online) 3D views of the complex–wavenumber–frequency solutions solved from the LOPE–based guided wave characteristic equation for a carbon–epoxy laminate with a layup of [0°/0°/0°]. (**a**) A 3D view showing the solutions for non–propagating (red) and forward and backward propagating (blue) guided wave modes. (**b**) A 3D view of dispersion curves in a domain with Re(*k*) > 0 and Im(*k*) > 0.

To better understand the propagating and non–propagating modes, the relationship between the complex wavenumber and frequency is studied. Figure 10 gives a 3D view of the complex–wavenumber–frequency solutions obtained by solving the LOPE–based characteristic equation for viscoelastic guided waves in a carbon–epoxy laminate with a layup of [0°/0°/0°]. The blue curves in Figure 10 represent propagating modes, and the red curves are for non–propagating modes. In Figure 10a, it is evident that the wavenumbers of propagating guided waves are distributed near the plane Im(*k*) = 0. In addition, the curves of propagating modes are symmetric about the plane Re(*k*) = 0, as our solutions consider both forward– and backward–propagating waves with Re(*k*) > 0 and Re(*k*) < 0, respectively. As shown in Figure 10b, when the frequency *f* varies from 0 to 5 MHz, the imaginary part Im(*k*) varies in a small range of 0 to 0.4 mm^−1^, and the real part Re(*k*) varies in a range from 0 to 15 mm^−1^. Moreover, it can be seen that the curves of high–order modes gradually extend to the imaginary wavenumber space with the frequency decrease.

### 4.6. Mode Shapes of Guided Waves

**Figure 11:**
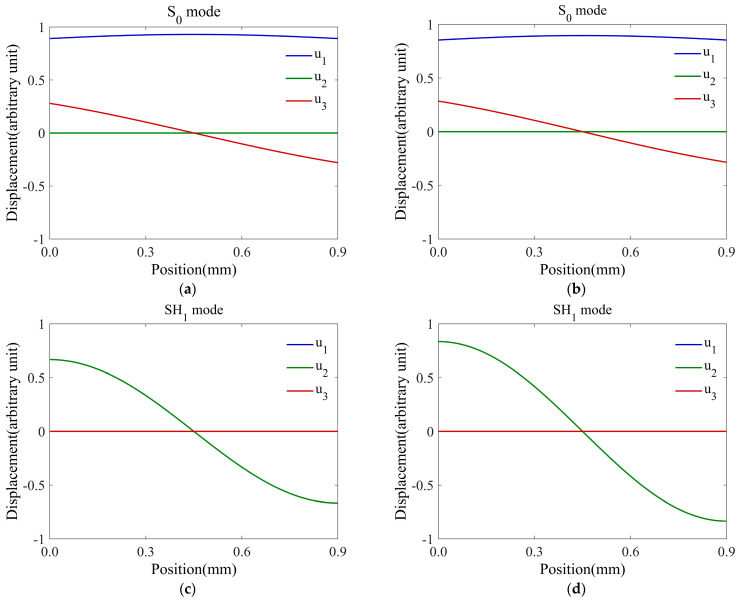
(Color online) Displacement mode shapes for the fundamental S_0_ mode at (**a**) 820 kHz and (**b**) 850 kHz, as well as the SH_1_ mode at (**c**) 820 kHz and (**d**) 850 kHz.

In order to elucidate the intrinsic mechanism of the curve–tracing strategy in Section 3.3, this subsection calculates the mode shapes (i.e., displacement distributions versus depths) at points near the intersection of the two–phase velocity curves. For example, the point ‘a’ at 830 kHz shown in Figure 9a is an intersection of the S_0_ and SH_1_ phase–velocity curves. Figure 11 reveals the mode shapes of both the S_0_ and SH_1_ modes at 820 kHz and 850 kHz on the left and right sides of the curve crossing point. From Figure 11a,b, it can be seen that the displacement component $u_{1}$ for the S_0_ mode in the *x*_1_ direction is significantly larger than the displacement components $u_{2}$ and $u_{3}$. Meanwhile, the in–plane displacement component $u_{1}$ is symmetric about the central plane of the three–ply composite, and the out–of–plane displacement $u_{3}$ is antisymmetric with respect to the central plane, agreeing with the typical mode shape characteristics of the S_0_ mode. The displacement distributions of the SH_1_ mode at 820 and 850 kHz are shown in Figure 11c,d, respectively. Both plots satisfy the typical mode shape characteristics of the SH_1_ mode. From Figure 11, it can be seen that the mode shapes of S_0_ and SH_1_ are dissimilar, although their phase–velocity–frequency points are very close to each other. Moreover, as the frequency increases from 820 and 850 kHz, the changes in their displacement amplitudes are different due to their different attenuation–frequency relations.

## 5. Conclusions

This study investigates guided wave properties such as phase–velocity–frequency dispersion relations, attenuation–frequency relations, and mode shapes in anisotropic laminated composites with the viscoelastic properties considered. Particularly, we formulated the characteristic equation of viscoelastic guide waves by leveraging the LOPE–based mode shape approximation and the hysteresis viscoelastic model–based viscoelastodynamics. Then, we solved the complex–wavenumber–frequency solutions of the characteristic equation using an improved root–finding algorithm leveraging both the coefficient matrix determinant ratios and local tracking search intervals. Numerical studies show that our approach can solve the frequency–complex–wavenumber relations for viscoelastic guided waves, including both the propagating and non–propagating waves, for viscoelastic anisotropic CFRP composites.

Based on the presented theoretical model, we investigated the effects of viscosity and fiber orientation on the phase–velocity and attenuation curves for viscoelastic guided waves in anisotropic carbon–epoxy laminates. Our results show that the viscosity in the hysteresis model mainly affects the frequency–dependent attenuation of viscoelastic guided waves. For the fundamental A_0_ and SH_0_ guided wave modes, the attenuation values increase nearly linearly as the frequency increases. The attenuation values of the S_0_ modes show nearly linear trends with the frequency increase, except for the sharp peaks shown in Figure 8f. Compared to viscosity, which mainly affects wave attenuation, the fiber orientation influences both the phase–velocity and attenuation curves. In the future, we plan to improve the LOPE–based model in this work to include both the pre–stress and viscoelastic effects. We also plan to leverage the dispersion curves solved by the LOPE–based model to assist in the development of guided wave–based methods for detecting defects in viscoelastic materials, as well as monitoring the composite curing process. We expect the theoretical work on viscoelastic guided waves to be beneficial for the development of guided wave–based techniques for the NDT and SHM of viscoelastic anisotropic laminated composites.

## Figures and Tables

**Table 1:** Material properties of an aluminum plate and a unidirectional T300/914 CFRP lamina. Units: *C*_ij_ (GPa), *μ*_ij_ (GPa), *ρ* (g/cm^3^).

Property	*c*_11_	*c*_12_	*c*_13_	*c*_22_	*c*_23_	*c*_33_	*c*_44_	*c*_55_	*c*_66_	*ρ*
Aluminum	92.88	39.81	39.81	92.88	39.81	92.88	26.54	26.54	26.54	2.7
T300/914	143.8	6.2	6.2	13.3	6.5	13.3	3.6	5.7	5.7	1.56

**Table 2:** Viscoelastic properties of a carbon–epoxy plate for the comparison study. Units: *C*ij (GPa), *μ*ij (GPa), and *ρ* (g/cm^3^).

*c*_11_	*c*_12_	*c*_13_	*c*_22_	*c*_23_	*c*_33_	*c*_44_	*c*_55_	*c*_66_	*ρ*
86.6	9	6.4	13.5	6.8	14	2.72	4.06	4.7	1.56
*μ*_11_	*μ*_12_	*μ*_13_	*μ*_22_	*μ*_23_	*μ*_33_	*μ*_44_	*μ*_55_	*μ*_66_	
7.50	0.30	0.60	0.60	0.25	0.28	0.10	0.12	0.28	

**Table 3:** Elastic and viscous constants for a viscoelastic lamina used in the carbon–epoxy laminated composite. Units: *C*_ij_ (GPa), *μ*_ij_ (GPa), *ρ* (g/cm^3^).

*c*_11_	*c*_12_	*c*_13_	*c*_22_	*c*_23_	*c*_33_	*c*_44_	*c*_55_	*c*_66_	*ρ*
132	6.9	12.3	5.9	5.5	12.1	3.32	6.21	6.15	1.56
*μ*_11_	*μ*_12_	*μ*_13_	*μ*_22_	*μ*_23_	*μ*_33_	*μ*_44_	*μ*_55_	*μ*_66_	
0.400	0.001	0.016	0.037	0.021	0.043	0.009	0.015	0.020	

## Data Availability

The authors declare that all data supporting the findings of this study are available within the article. Further information is available from the corresponding author upon reasonable request.

## Appendix A

The matrix elements $A_{\alpha \beta }^{j,m}$ and $M_{m}^{j}$ in Equation (14) are shown as below.

$$A_{11}^{m,j}=C_{55}u\left(m,j,0,2\right)+{\left(ik\right)}^{2}C_{11}u\left(m,j,0,1\right)+C_{55}K\left(m,j,0,1\right)$$

$$A_{12}^{m,j}=c_{45}u\left(m,j,0,1\right)+{\left(ik\right)}^{2}c_{16}u\left(m,j,0,0\right)+c_{45}K\left(m,j,0,1\right)$$

$$A_{13}^{m,j}=ik\left(c_{55}+c_{13}\right)u\left(m,j,0,1\right)+\left(ik\right)c_{55}K\left(m,j,0,0\right)$$

$$A_{21}^{m,j}=c_{45}u\left(m,j,0,2\right)+{\left(ik\right)}^{2}c_{16}u\left(m,j,0,0\right)+c_{45}K\left(m,j,0,1\right)$$

$$A_{22}^{m,j}=c_{44}u\left(m,j,0,2\right)+{\left(ik\right)}^{2}c_{66}u\left(m,j,0,0\right)+c_{44}K\left(m,j,0,1\right)$$

$$A_{23}^{m,j}=ik(c_{45}+c_{36})u\left(m,j,0,1\right)+ikc_{45}K\left(m,j,0,0\right)$$

$$A_{31}^{m,j}=ik\left(c_{55}+c_{13}\right)u\left(m,j,0,1\right)+\left(ik\right)c_{13}K\left(m,j,0,0\right)$$

$$A_{32}^{m,j}=ik(c_{45}+c_{36})u\left(m,j,0,1\right)+\left(ik\right)c_{36}K\left(m,j,0,0\right)$$

$$A_{33}^{m,j}=c_{33}u\left(m,j,0,2\right)+{\left(ik\right)}^{2}c_{55}u\left(m,j,0,0\right)+c_{33}K\left(m,j,0,0\right)$$

$$M_{j}^{m}=u\left(m,j,0,0\right)$$

$$u\left(m,j,n,l\right)=\int_{0}^{h}Q_{m}^{*}\left(x_{3}\right)\cdot x_{3}^{n}\cdot \frac{\partial^{l}Q_{n}\left(x_{3}\right)}{\partial x_{3}^{l}}dx_{3}$$

$$K\left(m,j,n,l\right)=\int_{-\infty }^{+\infty }Q_{m}^{*}\left(x_{3}\right)\cdot x_{3}^{n}\cdot \frac{\partial^{l}Q_{n}\left(x_{3}\right)}{\partial x_{3}^{l}}\cdot \frac{\partial \left[\pi \left(x_{3}-a\right)-\pi \left(x_{3}-b\right)\right]}{\partial x_{3}}dx_{3}$$

## References

[1] Goh G.D., Yap Y.L., Agarwala S., Yeong W.Y. (2018). Recent progress in additive manufacturing of fiber reinforced polymer composite. Adv. Mater. Technol..

[2] Shi L.L., Song G.J., Li P.Y., Li X.R., Pan D., Huang Y.D., Ma L.C., Guo Z.H. (2021). Enhancing interfacial performance of epoxy resin composites via in–situ nucleophilic addition polymerization modification of carbon fibers with hyperbranched polyimidazole. Compos. Sci. Technol..

[3] Chu S.H., Li L.G., Kwan A.K.H. (2021). Development of extrudable high strength fiber reinforced concrete incorporating nano calcium carbonate. Addit. Manuf..

[4] Withers P.J. (2007). Mapping residual and internal stress in materials by neutron diffraction. Comptes Rendus Phys..

[5] Li C., Xian G., Li H. (2019). Effect of postcuring immersed in water under hydraulic pressure on fatigue performance of large–diameter pultruded carbon/glass hybrid rod. Fatigue Fract. Eng. Mater. Struct..

[6] Panella F.W., Pirinu A. (2021). Fatigue and damage analysis on aeronautical CFRP elements under tension and bending loads: Two cases of study. Int. J. Fatigue.

[7] Xian G., Guo R., Li C., Wang Y. (2022). Mechanical performance evolution and life prediction of prestressed CFRP plate exposed to hygrothermal and freeze–thaw environments. Compos. Struct..

[8] Pan E., Rogers J., Datta S.K., Shah A.H. (1999). Mode selection of guided waves for ultrasonic inspection of gas pipelines with thick coating. Mech. Mater..

[9] Simonetti F. (2004). Lamb wave propagation in elastic plates coated with viscoelastic materials. J. Acoust. Soc. Am..

[10] Barshinger J.N., Rose J.L. (2004). Guided wave propagation in an elastic hollow cylinder coated with a viscoelastic material. IEEE Trans. Ultrason. Ferroelectr. Freq. Control..

[11] Huber A.M.A. (2023). Classification of solutions for guided waves in fluid–loaded viscoelastic composites with large numbers of layers. J. Acoust. Soc. Am..

[12] Marzani A. (2008). Time–transient response for ultrasonic guided waves propagating in damped cylinders. Int. J. Solids Struct..

[13] Luo Y., Li H., Xu B.Q., Xu G.D. (2010). Spectral finite element method modeling of ultrasonic guided waves propagation in layered viscoelastic film/substrate materials. J. Appl. Phys..

[14] Bause F., Gravenkamp H., Rautenberg J., Henning B. (2015). Transient modeling of ultrasonic guided waves in circular viscoelastic waveguides for inverse material characterization. Meas. Sci. Technol..

[15] Hayashi T., Song W., Rose J. (2003). Guided wave dispersion curves for a bar with an arbitrary cross–section, a rod and rail example. Ultrasonics.

[16] Bartoli I., Marzani A., Matt H., Lanza di Scalea F., Viola E. (2006). Modeling wave propagation in damped waveguides of arbitrary cross–section. J. Sound Vib..

[17] Mazzotti M., Marzani A., Bartoli I., Viola E. (2012). Guided waves dispersion analysis of prestressed viscoelastic waveguides by means of the SAFE method. Int. J. Solids Struct..

[18] Mazzotti M., Bartoli I., Marzani A., Viola E. (2013). A coupled SAFE–2.5D BEM approach for the dispersion analysis of damped leaky guided waves in embedded waveguides of arbitrary cross–section. Ultrasonics.

[19] Mazzotti M., Miniaci M., Bartoli I. (2019). A numerical method for modeling ultrasonic guided waves in thin–walled waveguides coupled to fluids. Comput Struct.

[20] Castaings M., Hosten B. (2003). Guided waves propagating in sandwich structures made of anisotropic, viscoelastic, composite materials. J. Acoust. Soc. Am..

[21] Quintanilla F.H., Fan Z., Lowe M.J.S., Craster R.V. (2015). Guided waves’ dispersion curves in anisotropic viscoelastic single– and multi–layered media. Proc. R. Soc. A.

[22] Torres-Arredondo M.A., Fritzen C.P. (2011). A viscoelastic plate theory for the fast modelling of Lamb wave solutions in NDT/SHM applications. Ultrasound.

[23] Yang X.D., Yu J.G. (2014). Viscoelastic Circumferential SH Wave in Graded Hollow Cylinders. Appl. Mech. Mater..

[24] Dahmen S., Amor M.B., Ghozlen M. (2016). Investigation of the coupled Lamb waves propagation in viscoelastic and anisotropic multilayer composites by Legendre polynomial method. Compos. Struct..

[25] Othmani C., Dahmen S., Njeh A., Ghozlen M. (2016). Investigation of guided waves propagation in orthotropic viscoelastic carbon–epoxy plate by Legendre polynomial method. Mech. Res. Commun..

[26] Liu H.Y., Liu S., Chen X., Lyu Y., Liu Z.H. (2020). Coupled Lamb waves propagation along the direction of non–principal symmetry axes in pre–stressed anisotropic composite lamina. Wave Motion.

[27] Li Z., Yu J., Zhang X., Zhang B., Liu Y., Li Z. (2024). Investigation on guided wave characteristics of prestressed bolts based on mechanics of incremental deformations theory. Measurement.

[28] Rhee S.H., Lee J.K., Lee J.J. (2007). The group velocity variation of Lamb wave in fiber reinforced composite plate. Ultrasonics.

[29] Yu J., Ratolojanahary F.E., Lefebvre J.E. (2011). Guided waves in functionally graded viscoelastic plates. Compos. Struct..

[30] Zhu F., Wang B., Qian Z., Pan E. (2018). Accurate characterization of 3D dispersion curves and mode shapes of waves propagating in generally anisotropic viscoelastic/elastic plates. Int. J. Solids. Struct..

[31] Orta A.H., Kersemans M., Abeele K.V.D. (2022). A comparative study for calculating dispersion curves in viscoelastic multi–layered plates. Compos. Struct..

[32] Wang L., Yuan F.G. (2007). Group velocity and characteristic wave curves of Lamb waves in composite: Modeling and experiments. Compos. Sci. Technol..

[33] He C., Liu H., Liu Z., Wu B. (2013). The propagation of coupled Lamb waves in multilayered arbitrary anisotropic composite laminates. J. Sound Vib..

